# Role of *Ttca* of *Citrobacter Werkmanii* in Bacterial Growth, Biocides Resistance, Biofilm Formation and Swimming Motility

**DOI:** 10.3390/ijms19092644

**Published:** 2018-09-06

**Authors:** Gang Zhou, Ying-Si Wang, Hong Peng, Xiao-Mo Huang, Xiao-Bao Xie, Qing-Shan Shi

**Affiliations:** Guangdong Open Laboratory of Applied Microbiology, Guangdong Provincial Key Laboratory of Microbial Culture Collection and Application, State Key Laboratory of Applied Microbiology Southern China, Guangdong Institute of Microbiology, Guangzhou, Guangdong 510070, China; zgbees@gdim.cn (G.Z.); wongvincy@163.com (Y.-S.W.); tangph163@163.com (H.P.); xmhuang@gdim.cn (X.-M.H.)

**Keywords:** *Citrobacter werkmanii*, biofilm formation, *ttcA* gene, Tn5 mutagenesis, swimming motility

## Abstract

To screen, identify and study the genes involved in isothiazolone resistance and biofilm formation in *Citrobacter werkmanii* strain BF-6. A Tn5 transposon library of approximately 900 mutants of *C. werkmanii* strain BF-6 was generated and screened to isolate 1,2-benzisothiazolin-3-one (BIT) resistant strains. In addition, the tRNA 2-thiocytidine (32) synthetase gene (*ttcA*) was deleted through homologous recombination and the resulting phenotypic changes of the *ΔttcA* mutant were studied. A total of 3 genes were successfully identified, among which *ΔttcA* mutant exhibited a reduction in growth rate and swimming motility. On the other hand, an increase in biofilms formation in *ΔttcA* were observed but not with a significant resistance enhancement to BIT. This work, for the first time, highlights the role of *ttcA* gene of *C. werkmanii* strain BF-6 in BIT resistance and biofilm formation.

## 1. Introduction

The isothiazolones have been proven for their high efficacy against many fungal and bacterial species [1,2]. Their mechanism of action includes the disruption of the metabolic pathways, inhibition of growth, respiration and energy generation, the destruction of protein thiols and production of free radicals [3]. Our previous study has reported that nitrogen metabolism and oxidative phosphorylation contributed to isothiazolone resistance in *Pseudomonas aeruginosa* [4]. Likewise, a few outer membrane proteins and genes responsible for peroxide-sensing in *Burkholderia cepacia* were shown to be involved in the same resistance [5]. 1,2-benzisothiazolin-3-one (BIT), a typical representative of isothiazolones, is an extremely effective biocidal agent often used as a preservative for controlling microbial growth and biofouling in a variety of cosmetic preparations and industrial water treatment applications. However, the complete and precise molecular mechanisms behind the bacterial resistance to isothiazolone, especially for BIT, remain elusive.

The genus *Citrobacter*, belonging to the family *Enterobacteriaceae*, is a distinct group of aerobic, Gram-negative, non-spore forming, rod-shaped bacteria [6]. Though commonly found in water, soil, food and intestinal tracts of animals and humans, some strains of this genus can cause serious opportunistic urinary and respiratory tracts infections [7]. In addition, *Citrobacter* sp. has been associated with extra intestinal disorders and enteric diseases [8,9]. Contrarily, numerous reports have shown the use of biofilm-immobilized *Citrobacter* sp. to combat environmental pollution or to produce biological metabolites [10,11]. Through our previous study, we have isolated a strain of *Citrobacter werkmanii* BF-6 from industrial waste in our laboratory, exhibiting a high capacity for biofilm formation. Interestingly, the processes and the resulting biofilms were found to be affected by various antimicrobial agents, especially by isothiazolones [12,13]. However, the underlying molecular mechanisms have not been elucidated.

In the present study, we have constructed a Tn5 insertion library of *C. werkmanii* BF-6 and successfully screened three BIT-resistant strains. One of the sites inserted by Tn5 transposon was the tRNA 2-thiocytidine (32) synthetase (*ttcA*) gene. With its deletion, the resulting phenotypic analysis identified the involvement of *ttcA* gene in the growth rate, BIT resistance, biofilm formation and swimming motility of *C. werkmanii* BF-6.

## 2. Results

### 2.1. Identification of the Insertion Sites of Tn5

**Figure 1:**
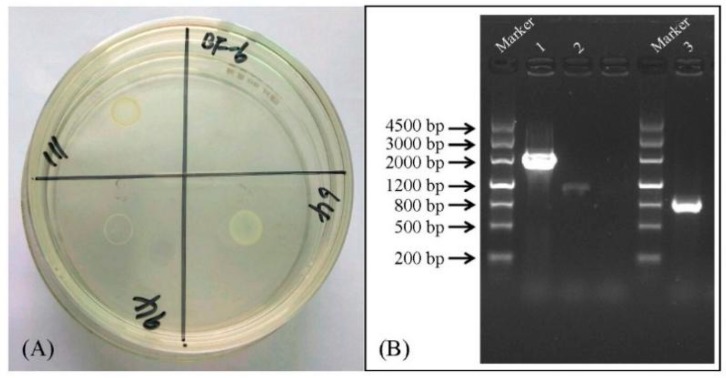
Screening of random resistant mutants using a LB plate supplemented with 32 mg/L BIT (**A**) and identification of *ΔttcA* and *ΔttcA*-com through PCR (**B**). Lane 1: *C. werkmanii* BF-6 (Control); Lane 2: *ΔttcA*; Lane 3: *ΔttcA*-com.

To identify the genes contributing to BIT-resistance, a random mutant library of *C. werkmanii* BF-6 was constructed using the plasmid pRL27 containing a Tn5 transposon. While screening for BIT-resistant clones, 3 out of 900 mutants exhibited significant growths as compared to wild type *C. werkmanii* BF-6 (Figure 1A). Successfully identified through the method of inverse PCR, the inserted genes were found to be: tRNA 2-thiocytidine (32) synthetase (*ttcA*), LysR family transcriptional regulator and HNH endonuclease (Table 1). In addition, a *ttcA* gene deletion strain (*ΔttcA*) was successfully constructed through homologous recombination and a complementary strain of *ΔttcA*-com was created using the plasmid pSRK-GM and the recipient *ΔttcA*. The PCR results for the identification of *ΔttcA* and *ΔttcA*-com using primers ttcA-WJ-F/R and ttcA-BS-F/R (Table 2) are shown in Figure 1B.

### 2.2. Characterization of ttcA Gene

**Figure 2:**
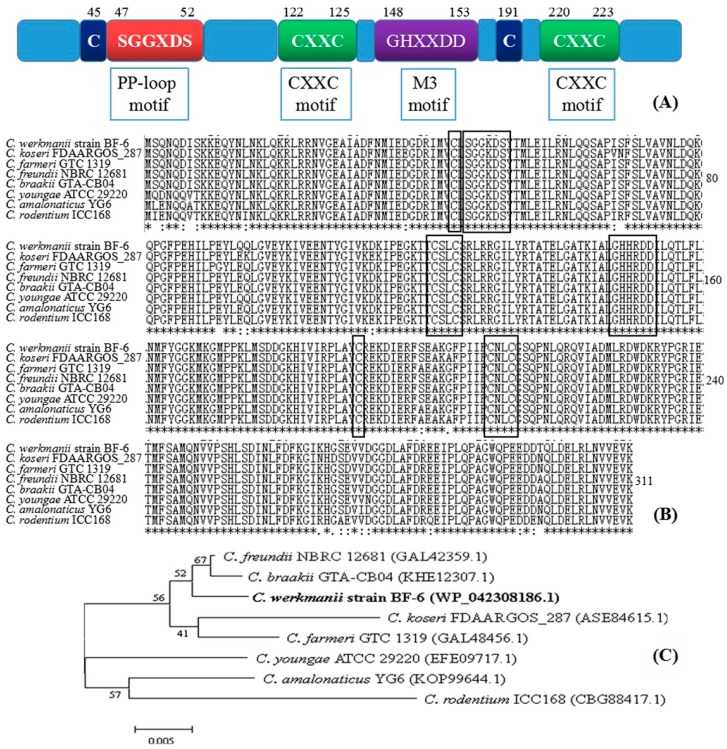
Analysis of the *ttcA* gene in *C. werkmanii* BF-6 and encoded protein sequence. Schematic representation of the TtcA protein with its conserved motifs (**A**). Sequence alignment of reported TtcA proteins representatives in the genus *Citrobacter* (**B**). The alignments were conducted with BioEdit software and the conserved amino acid residues and motifs are framed. Phylogenetic tree of TtcA protein of *C. werkmanii* BF-6 with the species of the same genus (**C**). This tree was constructed using bootstrapping and Neighbor-Joining methods with MEGA 4.0.

Among the 3 mutants, we found that the mutant numbered 64 on BIT plates displayed the best growth and thereby exhibited the strongest resistance to BIT (Figure 1A). The site of insertion for this mutant was identified to be tRNA 2-thiocytidine (32) synthetase (*ttcA*, Table 1). In order to elucidate the molecular function of *ttcA*, we first analyzed the gene structure and constructed a phylogenetic tree using MEGA 4.0. As shown in Figure 2A, there are four typical motifs present in protein TtcA: PP-loop motif (SGGXDS), M3 motif (GHXXDD), two CXXC motifs. Comparing the TtcA protein sequence of *C. werkmanii* BF-6 with other reported proteins sequences downloaded from NCBI database revealed the presence of the same motifs, thereby suggesting their conservation across the genus *Citrobacter* (Figure 2B). However, despite the same motifs in their protein sequences, the evolutionary distances of TtcA in the genus *Citrobacter* were not found to be close (Figure 2C).

### 2.3. Effect of BIT on Bacterial Growth

**Figure 3:**
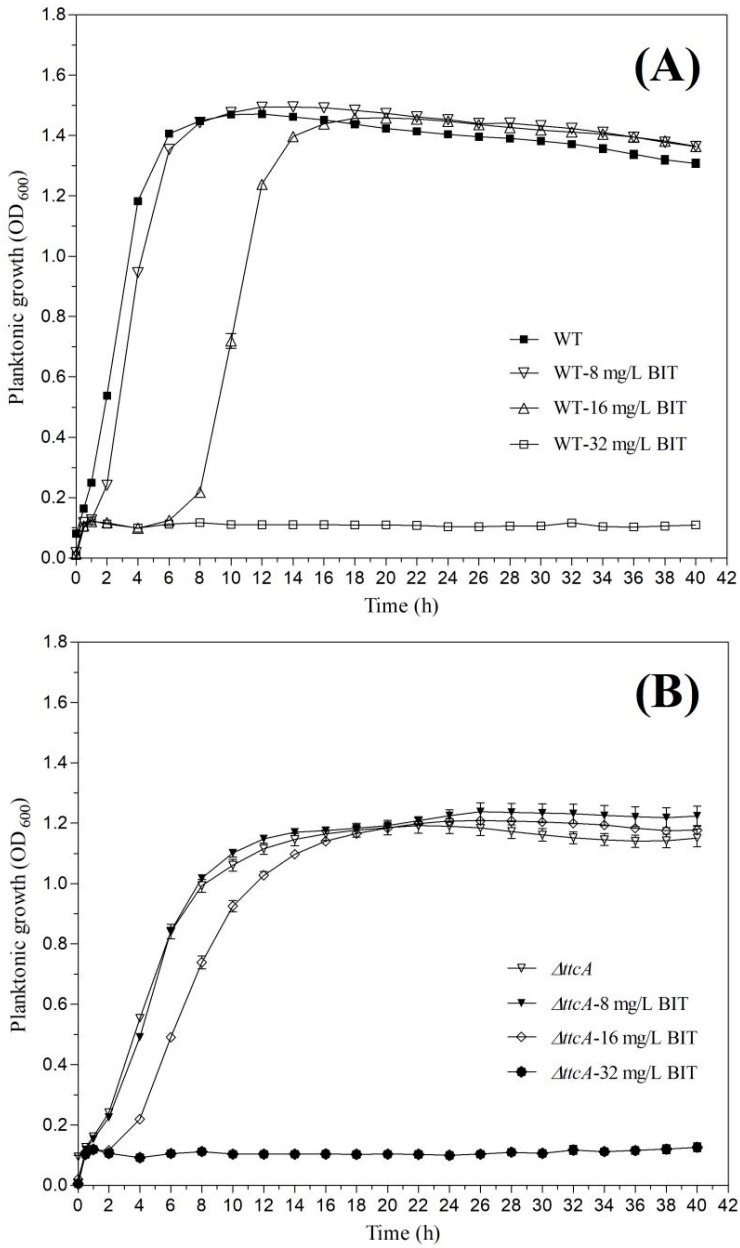

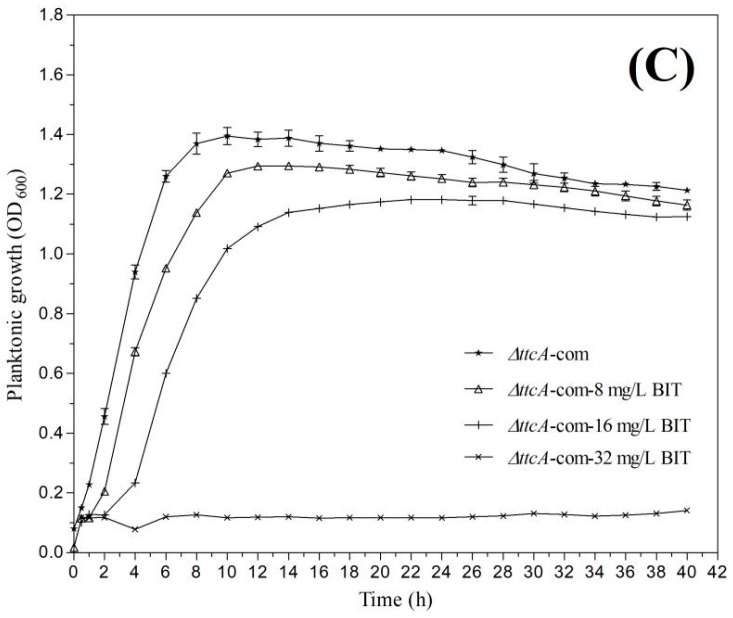
Growth curves of *C. werkmanii* BF-6 (**A**), *ΔttcA* (**B**) and *ΔttcA*-com (**C**). The strains were cultivated in LB medium supplemented with 0, 8, 16 or 32 mg/L BIT and incubated at 30 °C for 40 h.

The analysis of growth patterns of *C. werkmanii* BF-6, *ΔttcA* and *ΔttcA*-com in the absence or presence of BIT demonstrated that the growth rates were inhibited in all the three strains with an increase in concentration of BIT (Figure 3). No growth delays were observed in the presence of 8 mg/L BIT except in the case of *ΔttcA*-com. However, a dosage of 16 mg/L of BIT caused a notable growth delay in all three strains and *ΔttcA* exhibited the fastest speed to reach the exponential growth phase with a lower plateau growth as compared to wild type *C. werkmanii* BF-6 (Figure 3B). Further, the growths of *C. werkmanii* BF-6, *ΔttcA* and *ΔttcA*-com were found to be completely repressed by 32 mg/L of BIT (Figure 3).

### 2.4. Effect of BIT on Planktonic Growth and Initial Biofilm Formation

**Figure 4:**
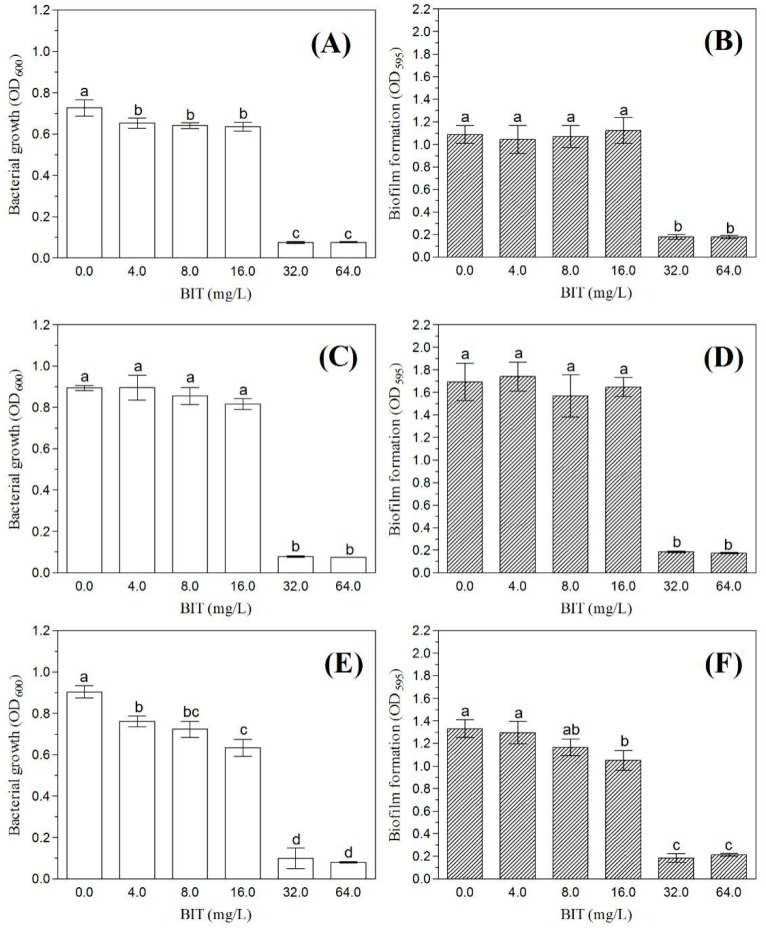
Planktonic growth and initial biofilm formation of *C. werkmanii* BF-6 (**A**,**B**), *ΔttcA* (**C**,**D**) and *ΔttcA*-com (**E**,**F**), respectively. The strains were grown in LB medium supplemented with indicted concentrations of BIT. All assays were conducted in triplicates and the columns marked with different letters (a, b, c or d) indicate that they are significantly different from each other according to Fisher’s LSD test (*p* < 0.05), calculated with the DPS data processing software.

The formation of bacterial biofilms can induce a declined susceptibility of certain bacteria to antimicrobial agents. Therefore, we detected the difference in the abilities of *C. werkmanii* BF-6, *ΔttcA* and *ΔttcA*-com to form biofilms in the presence of BIT. The results demonstrated that initial biofilm formations (including planktonic growth) were inhibited by BIT in a concentration-dependent manner (Figure 4). For both *C. werkmanii* BF-6 and *ΔttcA*, the biofilms formed in the presence of 4, 8 and 16 mg/L BIT were found to be same as their respective controls (Figure 4). However, *ΔttcA* was found to form more biofilms as compared to either *C. werkmanii* BF-6 or *ΔttcA*-com at each detected concentration of BIT (*p* < 0.05). In addition, the growth of planktonic cells and biofilms of *C. werkmanii* BF-6, *ΔttcA* and *ΔttcA*-com were almost completely inhibited by BIT at concentrations of 32 and 64 mg/L (Figure 4).

### 2.5. Role of ttcA in Bacterial Motility

**Figure 5:**
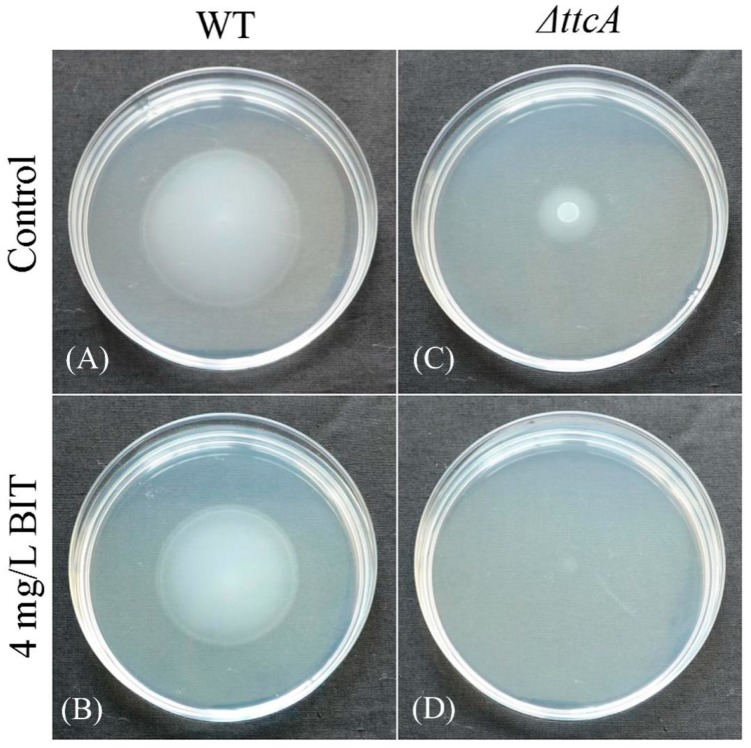
Swimming motility of *C. werkmanii* BF-6 (**A**,**B**) and *ΔttcA* (**C**,**D**) on semisolid agar plates supplemented with 0 or 4 mg/L BIT. Aliquots of 5 μL suspensions were inoculated at the center of the plate with a micropipette and then cultured for 24 h at 30 °C.

Swimming motility is considered as a flagellum-dependent form of movement observed in some Gram-negative bacteria. In the present study, the swimming abilities of *C. werkmanii* BF-6 and *ΔttcA* were determined and compared with each other. Results showed that the diameter of *ΔttcA* colony reduced by about 66.7% as compared with that of *C. werkmanii* BF-6 (Figure 5C) and the ability to swim by *ΔttcA* was almost found to be lost in the presence of 4 mg/L BIT (Figure 5D), while BF-6 lost approximately 16.7% of its swimming colony diameter at the same BIT concentration (Figure 5B). In addition, both BF-6 and *ΔttcA* were found to entirely lose their swimming motility when the BIT concentrations exceeded 8 mg/L (data not shown). Collectively, these results suggest that *ttcA* gene is necessary for the swimming motility of *C. werkmanii* while the anti-microbiological agent BIT could inhibit the same.

### 2.6. Effect of BIT on Biofilm Architecture

**Figure 6:**
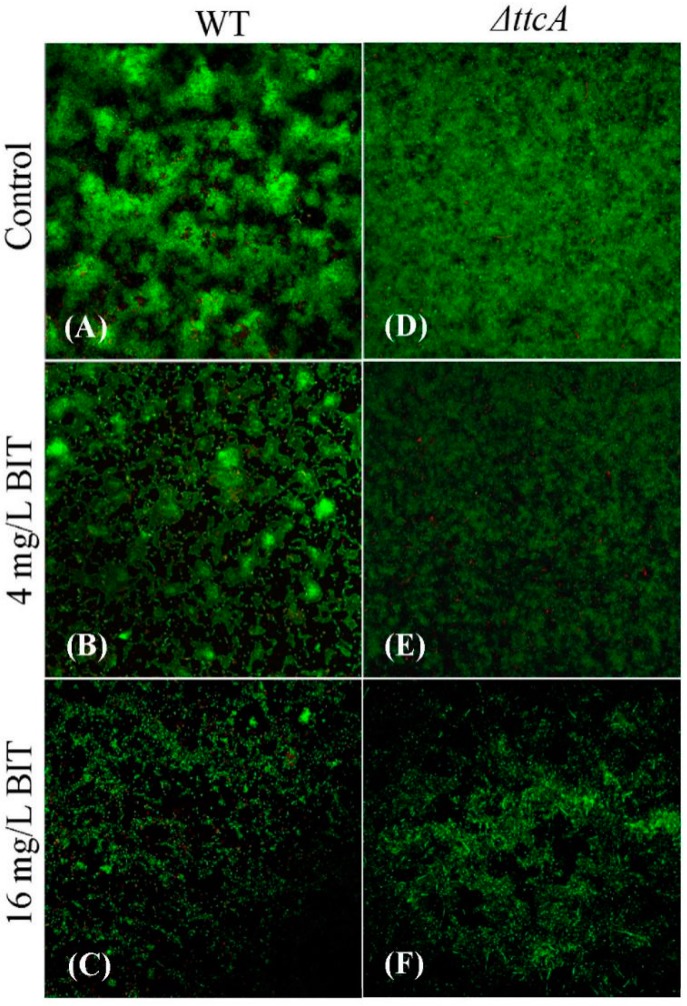
CLSM images of 4 days old biofilms of *C. werkmanii* BF-6 and *ΔttcA* in the presence of 0 (**A**,**D**), 4 (**B**,**E**) or 16 (**C**,**F**) mg/L BIT, respectively. The biofilms were cultured on the cover slides and stained with 5 μM SYTO9 dye and 30 μM propidium iodide. Magnification: 400×.

Since biofilm formations of *C. werkmanii* BF-6 and *ΔttcA* were found to be influenced by BIT, we speculated its effect on biofilm architectures too. In this regard, CLSM images of *C. werkmanii* BF-6 and *ΔttcA* biofilms in the absence or presence of BIT were analyzed to quantitatively calculate the parameters of their topographies and architectures. When cultured for 4 days, typical biofilms were formed by *C. werkmanii* BF-6 and *ΔttcA* on the surfaces of their respective glass slides in LB medium without BIT (Figure 6A,D). However, *ΔttcA* formed smoother biofilms and displayed a higher coverage when compared to *C. werkmanii* BF-6 (Figure 6). When supplemented with 4 and 16 mg/L of BIT, the biofilms of *C. werkmanii* BF-6 alone were seen to become sparse in nature (Figure 6). Upon further analysis of the CLSM acquired images using the COMSTAT 2.1 software, we noticed that the biofilm parameters comprising of average and maximum thickness, total biomass and average colony size were all higher in *ΔttcA* than in *C. werkmanii* BF-6, except for roughness coefficient, in all detected conditions (Table 3). These observations were consistent with the data obtained from CLSM.

## 3. Discussion

It has been reported that isothiazolones are capable of interacting oxidatively with the accessible thiols, such as glutathione or cysteine, within the microbial cells and thus lead to the inhibition of their metabolism and loss of viability [3,14]. Further, it has been proven that certain growth factor receptors and fibroblast cells along with several intracellular enzymes including transferases, oxidoreductases, isomerases, ligases and hydrolases can be inhibited by these microbiocides [15]. In the present study, we created a Tn5 transposon library of *C. werkmanii* BF-6 to screen and isolate BIT resistant strains. During this process, the study of three random insertion sites identified from transposon mutants (Table 1) revealed that *ttcA* as one of the genes that garnered further investigation. It is interesting to note that among the translated protein sequence of *ttcA* gene, there are two CXXC motifs (Figure 1A), within which the Cys residues coordinate a [4Fe-4S] cluster essential for the thiolation activity [16]. Based on data from the growth curves and resistance determination experiments (Figure 3 and Figure 4), we speculated that either these CXXC motifs or the internal cysteine (C, possesses S-H bond) could be the binding sites for BIT. However, it is important to note that neither the motif nor the protein is a unique binding site of BIT because the deletion of *ttcA* gene did not lead to the complete absence of BIT action (Figure 3 and Figure 4). Additionally, we found two CXXC motifs (CAYC and CAPC) in the protein sequence of HNH endonuclease (data not shown), which could also play vital roles in the binding of BIT and its subsequent development of resistance. However further study is needed to verify this hypothesis.

The protein tRNA 2-thiocytidine (32) synthetase (TtcA) is required for the thiolation of cytidine at position 32 of tRNA to form 2-thiocytidine (s(2)C32). The TtcA protein family is characterized by the presence of a PP-loop along with a CXXC motif in their central regions (Figure 2). Mutant analysis showed that the cysteines in this centrally conserved CXXC motif are essential for the formation of s(2)C32 [17]. It has been demonstrated that the TtcA enzymes in *Escherichia coli* contain a redox-active and oxygen-sensitive [4Fe-4S] cluster which are chelated by only three cysteine residues and are absolutely essential for their activity [16]. Meanwhile, it has also been proven that TtcA protein in *E. coli* is originally a tRNA-thiolating enzyme proceeding via an ATP-dependent pathway depending on a Fe-S cluster, as testified by in vivo and in vitro enzyme activity assays [16]. In another study, it was revealed that the deletion of *ttcA* gene in the genome of *Salmonella enterica* did not affect the growth rates of the *ΔttcA* mutants in comparison to their congenic wild type strains and that the lack of s(2)C32 did not cause any growth disadvantage in a mixed-population experiment [17]. However, we found that the inactivity of *ttcA* gene in *C. werkmanii* led to their delayed growth and lowered the growth curves (Figure 3) which suggest that the *ttcA* gene could be involved in different processes in different bacteria, with respect to their growth. In addition, the mutant strain was not isogenic with wild type *C. werkmanii* BF-6 and therefore could have different physical properties but the purpose of this manuscript was to determine the effect of the *ttcA* mutation, including all physical property changes.

Bacterial biofilms represent a life stage different from planktonic cells wherein individual cells are encased in a self-produced matrix of hydrated extracellular polymeric substances (EPS) consisting of polysaccharides, proteins, nucleic acids (eDNA) and lipids [18]. The EPS provide the mechanical stability in biofilms, mediating their adhesion to surfaces and forming a cohesive, three-dimensional polymer network that interconnects and transiently immobilizes biofilm cells [18]. In this study, observations through CLSM demonstrated that both BF-6 and *ΔttcA* could establish typical biofilms on covers slides (Figure 6). However, with *ΔttcA* displaying smoother biofilms with higher biomasses as compared to the wild type BF-6 (Table 3 and Figure 6), it can be suggested that *ttcA* gene is perhaps a negative regulatory factor for both biofilm formation and its structural organization in *C. werkmanii*.

It is known that the EPS matrix of biofilms can limit and further prevent the transport of antimicrobial agents to the cells by either reacting with the bactericides through either sorption, electrostatic or hydrophobic interactions, size exclusion or through the eventual degradation of biocides [19,20,21,22]. In this study, *ΔttcA* was seen to form denser biofilms as compared to *C. werkmanii* BF-6, which indicate a slight increase in resistance to BIT by *ΔttcA* (Figure 2 and Figure 6, Table 3). However, the barrier of biofilm to BIT only played a role at lower concentration of the drug, as higher dosages could still completely inhibit the growth of planktonic cells and biofilms.

Swimming motility is a flagellum-dependent form of movement observed in Gram-negative bacteria [23,24,25]. Through this study, we found that BIT could inhibit the swimming ability of *C. werkmanii*. Although the *ttcA* gene was verified to be essential for the swimming motility in this bacterium, the exact underlying mechanism needs to be elucidated.

## 4. Materials and Methods

### 4.1. Bacterial Strains and Chemicals

Wild type *C. werkmanii* BF-6 (WT) was previously isolated from industrial putrefaction in our laboratory [26] and cultures have been stocked at the Guangdong Culture Collection Center (Guangzhou, Guangdong, China) under the accession number GDMCC 1.1242. *Escherichia coli* S17-1(λpir)/pRL27 mini-Tn5-Km [27] and pYG4 were kind gifts from professors Lian-hui Zhang of South China Agricultural University, Guangzhou and Yan-guang Cong of Third Military Medical University, Chongqing, China, respectively. The above strains were routinely grown in Luria Bertani (LB) medium with or without the corresponding antibiotics (Kanamycin, 50 mg/L) at 30 °C or 37 °C for necessary durations. All chemicals used in this study were of reagent grade and purchased from Sigma (St. Louis, MO, USA), unless indicated otherwise.

### 4.2. Construction of Transposon for Mutagenesis

Mutagenesis was performed according to the method described previously, with slight modifications [28]. Both *E. coli* S17-1(λpir)/pRL27 mini-Tn5-Km and *C. werkmanii* BF-6 were cultured overnight at 37 °C in LB media in a constant-temperature shaker incubator set to 165 rpm. The donor *E. coli* S17-1(λpir)/pRL27 and the recipient *C. werkmanii* BF-6 were mixed in a ratio of 1:1 and then spotted on a LB agar plate which was covered with a 0.45 μm cellulose membrane. The plates were then cultured at 37 °C for at least 12 h to allow conjugational transfer of the plasmid pRL27 from the donor to the recipient cells. Following this, the co-cultivated bacteria on the membrane surface were washed and suspended in 2 mL of 10 mM MgSO_4_. About 100 μL of *C. werkmanii* BF-6 clones with the integrated Tn5 cassette were then spread onto LB agar plates supplemented with kanamycin. In addition, all the mutants were transferred onto LB plates supplemented with 32 mg/L BIT to screen for BIT-resistance.

### 4.3. Identification of Transposon Insertion Site

An inverse PCR method was employed to identify the flanking sequence of the inserted mini-Tn5 transposon in the genomes of the mutants [28]. Briefly, the total genomic DNAs of *C. werkmanii* BF-6 mutants were extracted from 1 mL of LB medium cultures using the EasyPure Bacteria Genomic DNA Kit (Transgen Biotech, Beijing, China). The isolated genomes were digested with TaqI (NEB, Beijing, China), self-ligated with T4 DNA ligase (NEB) and then used as templates for inverse PCR using the primers TnI-F and TnI-R (Table 2). The amplified fragments were separated by agarose gel electrophoresis, purified using the EasyPure Quick Gel Extraction Kit (Transgen Biotech, Beijing, China) and finally sequenced at the Invitrogen (Guangzhou, China). To verify the mutant genes, the obtained sequences were used to search the nucleotide databases developed by the National Center for Biotechnology Information (NCBI) and the available *C. werkmanii* BF-6 genome using the BLAST tool.

### 4.4. Construction of Gene Knockout Mutants and Complementary Strains

Upon analysis, we found that Tn5 was inserted into the tRNA 2-thiocytidine (32) synthetase gene (*ttcA*) and the resulting mutant exhibited an increased resistance to BIT. In order to testify the molecular role of *ttcA* in biocides resistance and biofilm formation, we deleted this gene from the genome of *C. werkmanii* BF-6. The gene knockout and complementation of *ttcA* was performed according to previously reported methods, with slight modifications [29]. Briefly, the flanking DNA fragments (1059 bp upstream with *Bgl*II site; 792 bp downstream with *Nde*I site) of the gene *ttcA* (locus_tag = “B2G73_RS05960”) were whole gene synthesized at GENEWIZ Biotechnology Co. Ltd. (Suzhou, China). The resulting fragment was digested with the enzymes *Bgl*II and *Nde*I (NEB) and then cloned into the pYG4 vector digested with the same endonucleases. The resulting plasmid of pYG4-ttcA was used to transform *E. coli* S17-1 cells and was subsequently mobilized into *C. werkmanii* BF-6 via biparental mating. The exconjugants were selected on LB medium containing kanamycin (50 mg/L) and rifampicin (100 mg/L) and the deletion mutant candidates were recovered on LB medium containing 5% sucrose and were finally confirmed by PCR using the primers ttcA-QJ-F/R (Table 2). To construct the complementary strain, the genomic DNA was first extracted from *C. werkmanii* BF-6 by using the EasyPure Genomic DNA Kit (Transgen Biotech, Beijing, China) following the manufacturer’s directions and the full-length *ttcA* gene with its upstream sequence was amplified using the primers ttcA-Com-F/R (Table 2). The PCR product was cloned into the shuttle vector pSRK-GM between the XbaI and HindIII sites. The recombinant plasmid pSRK-GM-Com was transferred into *E. coli* S17-1 cells and then subsequently mobilized into *C. werkmanii* BF-6 deletion strain of *ΔttcA*. The complemented strain was confirmed by PCR using the primers ttcA-BS-F/R (Table 2) and was designed as *ΔttcA*-com.

### 4.5. Determination of the Bacterial Growth Curves

The growth curves of *C. werkmanii* BF-6, *ΔttcA* and *ΔttcA*-com in the absence or presence of 0, 8, 16 and 32 mg/L BIT were determined in a total volume of 200 μL using a Bioscreen C microtiter plate (Labsystems, Helsinki, Finland), starting with an initial bacterial density of approximately 2 × 10^4^ cfu/mL. The cultures were incubated at 30 °C with constant shaking at medium speed for 40 h in a Bioscreen C analyzer (Labsystems, Helsinki, Finland) following the instructions in the manual and the bacterial growth was recorded every 30 min by measuring the optical density (OD) at 600 nm.

### 4.6. In Vitro Inhibitory Effect of BIT on Initial Biofilm Formation

A column of microtiter plate (Corning Inc., Corning, NY, USA) was inoculated with 100 μL of either BF-6, *ΔttcA* or *ΔttcA*-com suspensions (OD_600_ = 0.10). Dilutions of BIT stock solution in a total volume of 100 μL were added such that the final concentrations of BIT were as follows: 0, 4, 8, 16, 32 and 64 mg/L. Eight replicates were set for each drug concentration and the plates were transferred into a static incubator where they were cultured at 30 °C for 4 days. Following this, they were subsequently managed according to the previously described methods, with minor modifications [12,30]. The growth rates of BF-6, *ΔttcA* and *ΔttcA*-com planktonic cells in the cultured plates were measured using a Multiskan GO Reader (Thermo Scientific, Waltham, MA, USA) at 600 nm. The cultivation in each well was discarded gently and washed with 250 μL of sterile water for at least three times to completely remove any of the floating cells. After drying the plate for 30 min at room temperature, each well was filled with 250 μL of 0.1% crystal violet solution (Shanghai Chemical Reagents Co. Ltd., Shanghai, China) to stain the sessile cells attached on the walls of the well for about 40 min. Subsequently, excess of the staining dye was washed away using sterile water for at least three times. After drying the plate at room temperature again, the wells were filled with 250 μL of 95% ethanol (Shanghai Chemical Reagents Co. Ltd., Shanghai, China) to resolubilize the dye for at least 30 min. The OD_595_ of each well was determined using the same Reader. Meanwhile, the cultures without any BIT were considered as controls.

### 4.7. Motility Assay

The motility assay for *C. werkmanii* BF-6 and *ΔttcA* was determined according to the methods described previously, with slight modifications [24,31]. Briefly, several swimming plates (10 g/L tryptone, 5 g/L NaCl and 0.3% agar) were inoculated with 2 μL of bacterial suspensions (OD_600_ = 0.05) in the center of the plates, following which, they were transferred into an incubator set to 30 °C. After 24 h of incubation, the plates were taken out and the diameters of the colonies in each plate were measured using a digital Vernier caliper (CD-20CP, Kawasaki, Japan). Additionally, the plates were photographed using a digital camera (Nikon D7200, AF-S DX 18-140 mm; Nikon, Tokyo, Japan).

### 4.8. Observation of Biofilm Structures with Confocal Laser Scanning Microscopy (CLSM)

Biofilms of *C. werkmanii* BF-6 and *ΔttcA* were first allowed to form on pre-sterilized glass microscope slides and then observed with CLSM according to the method reported previously [32]. Briefly, aliquots of 2 mL of BF-6 or *ΔttcA* suspensions (OD_600_ = 0.05) supplemented with BIT to final concentrations of 0, 4 or 16 mg/L were added to a well of microtiter plate (12 wells, Corning, NJ, USA) containing a cover slip. Following an incubation under static conditions at 30 °C for 4 days, the glass slips were gently taken out and stained with 5 μM SYTO9 dye (Invitrogen, Carlsbad, CA, USA) and 30 μM propidium iodide (Sigma, St. Louis, MO, USA) in dark for at least 15 min at room temperature. Finally, the stained biofilms were visualized using CLSM (LSM 710 Zeiss, Jena, Germany). In addition, a quantitative analysis of biofilm parameters of the acquired images was analyzed using a COMSAT 2.1 software [33,34].

### 4.9. Statistical Analysis

All the data collected in this study were recorded as the mean ± standard deviation (SD) and were also subjected to one-way ANOVA test. This was followed by comparison of multiple treatment levels with the controls using Fisher’s LSD test. All the statistical calculations were done using a data processing system (DPS) software [35] and differences were considered statistically significant when *p* < 0.05.

## 5. Conclusions

Our results from the present study demonstrate that *ttcA* gene in *C. werkmanii* is involved in its planktonic growth, BIT resistance, swimming motility, initial biofilm formation and the underlying structural stability. *ttcA* plays a significant role in the thiolation of cytidine at position 32 of tRNA. This study, to the best of our knowledge, is the first attempt to construct a relationship between the *ttcA* gene with isothiazolone resistances, swimming motility and biofilm formation in *C. werkmanii*. However, the detailed molecular mechanisms of the biological processes demonstrated in this study warrant further research.

## Figures and Tables

**Table 1:** Identification of the random insertion sites of Tn5 transposon.

Number	Locus Tag	Gene Name	Description
64	B2G73_RS05960	*ttcA*	tRNA 2-thiocytidine(32) synthetase
94	B2G73_RS07535	-	LysR family transcriptional regulator
111	B2G73_RS05205	-	HNH endonuclease

“-”: no gene name.

**Table 2:** List of primers used for *ttcA* gene knockout and complementation.

Primer Names	Forward Primer Sequences (5′-3′)	Reverse Primer Sequences (5′-3′)	Application
TnI-F/R	GGAGAGGCTATTCGGCTATG	GTAAGGTGATCCGGTGGATG	Identification of Tn5 inserted sites
ttcA-WJ-F/R	ATTATCGCTTCTCACCGACCTG	AACGTCCAGCCACGGTAAAATC	Identification of *ttcA* deletion
ttcAP-F/R	GCTCTAGAGTGATGCATACTCTTCTGAT	CCCAAGCTTTTACTTCACTTCCACAACGT	*ttcA* complementation
ttcA-BS-F/R	CTTCAATATGATCGAAGACGGC	TTGGGTATCATCCTCTTCTGGC	Partial sequence of *ttcA* gene

**Table 3:** Quantitation of biofilm architecture.

Parameters *	WT (Mean ± SD)			*ΔttcA* (Mean ± SD)		
	0 mg/L BIT	4 mg/L BIT	16 mg/L BIT	0 mg/L BIT	4 mg/L BIT	16 mg/L BIT
Maximum thickness (μm)	12.00 ± 2.00	11.33 ± 1.15	8.00 ± 0.00	20.77 ± 2.69	18.00 ± 4.00	16.67 ± 2.31
Average thickness (μm)	8.26 ± 1.40	5.86 ± 1.17	3.54 ± 1.29	17.33 ± 2.31	9.58 ± 1.72	5.79 ± 1.27
Total biomass (μm^3^/μm^2^)	12.06 ± 0.98	5.58 ± 1.20	3.42 ± 1.06	14.52 ± 0.14	9.78 ± 1.63	6.14 ± 1.27
Average colony size (μm^2^)	195.67 ± 17.65	182.18 ± 12.12	59.00 ± 6.58	213.75 ± 7.93	192.55 ± 18.54	155.90 ± 17.86
Roughness coefficient	0.22 ± 0.02	0.51 ± 0.02	0.82 ± 0.21	0.08 ± 0.02	0.11 ± 0.01	0.36 ± 0.06

* Quantitation of biofilm parameters including maximum thickness, average thickness, total biomass, average colony size and roughness coefficient were evaluated using COMSTAT 2.1. The results are means of datasets obtained from the analysis of eight CLSM images acquired at random positions in each of the biofilms. Standard deviations (SD) are indicated along with the means.

## Abbreviations

BIT	1,2-benzisothiazolin-3-one
LB	Luria Bertani
CLSM	Confocal Laser Scanning Microscopy
